# Impact of Peer-Assisted Learning in Chest Tube Insertion Education on Surgical Residents

**DOI:** 10.30476/BEAT.2022.94348.1336

**Published:** 2022-04

**Authors:** Iman Deilamy, Mitra Amini, Hamid Reza Abbasi, Shahram Bolandparvaz, Shahram Paydar

**Affiliations:** 1 *Clinical Education Research Center, Shiraz University of Medical Sciences, Shiraz, Iran*; 2 *Department of Surgery, School of Medicine, Shiraz University of Medical Sciences, Shiraz, Iran*; 3 *Trauma Research Center, Rajaee (Emtiaz) Trauma Hospital, Shiraz University of Medical Sciences, Shiraz, Iran*

**Keywords:** Medical education, Chest tubes, Peer-assisted learning, Surgery residents

## Abstract

**Objective:**

To investigate the impact of peer-assisted learning (PAL) in chest tube insertion education on surgical residents.

**Methods:**

This study is a quasi-experimental study conducted on thirty general surgeon residents enrolled in the PAL program. They were divided into two learner groups (A and B) based on the period of residency start. Group A and B had six and one months of general surgery residency experience, respectively. All participants received adequate training for chest tube insertion by a recently graduated general surgeon. Chest tubes insertion skill was assessed using the tool for assessing chest tube insertion competency (TACTIC) test.

**Results:**

Post-TACTIC test score was significantly higher (*p*=0.001) than Pre-TACTIC test score in both groups. However, a comparison of mean Pre-TACTIC test scores and mean Post-TACTIC test scores between group A and group B showed that PAL effectiveness in group A was significantly higher (*p*=0.001) than group B.

**Conclusion:**

There was a positive relationship between the PAL program and the improvement of chest tube insertion technical skills in surgical residents. Based on our findings and similar studies, it can be concluded that the PAL program can increase the chest tube insertion skill of surgical residents.

## Introduction

The insertion of a chest tube is part of the surgeons’ routine activities that are typically based on knowledge, practice, experience, and judgment [1]. However, it may be associated with severe complications [2]. Several studies showed the need for a structured training program to minimize complications of chest tube insertion and enhance patient safety [3-5]. 

Recently, medical education has faced significant challenges in training new residents during the 2019 coronavirus pandemic (COVID-19) [6]. Virtual education is necessary to minimize teaching and assessment disruption [7]. However, using other learning strategies such as peer-assisted learning (PAL) can validate its impact on students’ active learning [8]. 

The LAP is a type of learning in which students assist one another to learn. Based on the Association of Medical Education in Europe (AMEE) guide, PAL offers about 18 different types of learning strategies that can be carried out by peers [9]. Peer education positively impacts both the teacher and the learners [10]. However, a reliable assessment tool is required to evaluate the mastery learning concept. 

Shefrin *et al*., developed a 40-point assessment tool with 20-item that scored from zero to two. This tool is the tool for assessing chest tube insertion competency (TACTIC). The TACTIC method demonstrated good inter-lateral reliability, content validity, and conceptual validity to assess a practitioner’s ability to insert chest tubes into a simulated setting [11]. The objective is to study the impact of peer learning in thoracic tube insertion teach on surgical residents using the TACTIC test.

## Materials and Methods

The study is a quasi-experimental study of a single center that was conducted on junior residents in general surgery at Shahid Rajaee Trauma Hospital, Shiraz University of Medical Sciences, Shiraz, Iran. Before the enrollment of residents, approval was received from the local ethics committee of the Shiraz University of Medical Sciences (IR.SUMS.REC.1399.1209). Participation in the research was entirely voluntary. We invited the general surgery residents to participate in two courses. From 33 students, 30 enrolled in the study. All participants were divided into two learner groups (A and B) based on the period of residency start. The A and B groups had six and one months of general surgery residency experience before the study, respectively. As educational policies demand in Shiraz University of Medical Education, residents who participated in the study after six months (group A) were educated by their peers on inserting chest tubes through observation and participation in surgical wards. But group B had no previous academic education.

On the workshop day, a pre-test was taken from both groups. Chest tubes insertion skill was assessed using the TACTIC tool. The students were directly instructed in chest tubes insertion by an ATLS instructor, a recently graduated general surgeon. The duration of the workshop was four hours, which was done in two parts: theoretical and practical. The theory part was included conference and video training. The videos were adapted from the New England Journal of Medicine (NEJM). The NEJM video was around 16 minutes in length. It provides an overview of the procedure’s steps, indications, and contraindications, required equipment, key procedural steps, and potential complications. Information is provided in structured form with graphics animations and overlays. The procedure is also performed using a cadaver model and an actual patient. The lecture was given by a certified advanced traumatology and resuscitation instructor for 17 minutes. It shows a small group of students with an instructor, an artificial model, and there is a bidirectional dialogue between the student and the instructor. All participants received adequate training for chest tube insertion using moulage. After a week, a post-test was taken from both groups.

Statistical analysis was performed by SPSS version 25 software (SPSS Inc., Chicago, IL, USA). Wilcoxon tests (a non-parametric statistical hypothesis test) were used for comparing the means of numerical data. In this test, if the *p*-value is less than 0.05, it shows a significant difference between the mean of samples in the two groups, and this statistic is confirmed with 95% confidence.

## Results

In this study, a peer leader (a recently graduated general surgeon) and 30 general surgery residents were enrolled. There were 15 residents in each of the two groups. All residents that enrolled completed the study. There were 19 (63.3%) men and 11 (36.7%) women among the learners. There were 11 (73.3%) men and 4 (26.7%) women in Group A, and there were 8 (53.3%) men, and 7 (46.7%) women in Group B. Group A and Group B had a mean age of 32 (range: 29-44) and 29.5 (range: 27-36) years, respectively. 

There was no significant difference between the score of the Pre-TACTIC test in the A and B groups. However, the score of the post-TACTIC test in Group A showed a significant difference (*p*=0.001) compared to Group B. Group A individual had a mean score of the Pre-TACTIC test, 22.86 (range: 14-40) compared to Group B with the mean score of 18.73 (range: 10-24). Comparing the Pre-TACTIC test score and Post-TACTIC test score in both groups showed a significant difference (*p*=0.001) (Table 1).

**Table 1:** Comparison the scores of the tool for assessing chest tube insertion competency (TACTIC) test in Group A and Group B

**Group**		**TACTIC test score**			**P-value**
		**Mean**	**Minimum**	**Maximum**	
A^a^	Pre-test	22.86	14	40	0.001
	Post-test	37	31	40	
B^b^	Pre-test	18.73	10	24	0.001
	Post-test	29.33	20	35	

^a^A: Students who had six months of general surgery residency experience before the study; ^b^B: Students who had one month of general surgery residency experience before the study.

## Discussion

The peer-assisted learning (PAL) is increasingly used in medical education and has become an integral part of modern medical programs [12]. PAL seems to be a perfect way to impact student participation, learning, and clinical performance [13]. Inserting a chest tube is one of the most common surgical procedures appropriately done by a trained general surgeon [14]. However, it can be associated with multiple complications that result exclusively from resident physicians. Insertional complications include visceral or parietal lesions of the intercostal artery or intraparenchymal lung, and positional complications include extrathoracic placement or atypical intrathoracic placement causing tube insufficiency and replacement [15]. Resident training has a critical role to reduce these complications [15]. 

This study used PAL to learn chest tube insertion for surgical residents by TACTIC test. Our results showed that PAL positively impacts general surgery residents’ chest tube insertion procedure skills. However, prolonged presence in the surgical ward played an essential role to have more skills than residents who spent less time in the surgical ward. With six months of experience in the surgical ward, group A had a higher mean score of Pre and post-TACTIC tests than the other group with one month experience. Comparison the mean Pre and post-TACTIC test scores between group A and group B showed that PAL effectiveness in group A was significantly higher (*p*= 0.001) than group B. Carey *et al*., showed that the PAL decreased challenges of clinical practice [16]. Pelloux *et al*., assessed the performance of the PAL relative to instructor-led instruction for peripheral venous catheter insertion training. Their results showed that PAL positively impacts peripheral venous catheter insertion training and can be as an effective as instructor-led instruction [17]. Bennett *et al*., showed that PAL is a practical and achievable method to teach surgical competence [18]. Varghese *et al*., confirm that PAL improves the learning experience and is beneficial [19]. Ribeiro *et al*.. assessed 34 students before and after the PAL program. They identified that the workshop score of students who obtained the peer assisted learning program was significantly higher than the workshop score of students before the PAL program. Their study demonstrated significant qualitative and quantitative improvement in technical knowledge and skills among students [20]. Kamble *et al*., assessed the benefits of PAL on learning of physiological basis of electrocardiography. Mean post-test scores were higher and statistically significant compared to the preliminary test in this study. The class average normalized gain for a post-test score was more than 70% better than the pre-test score. Accordingly, they identified that the PAL is a practical and an effective way to teach complex physiology concepts [21]. However, our study and several other studies showed the benefit of PAL; one of the major limitations of our study is its small sample size. In order to ensure that our results are generalizable to other forms of PAL across medical schools worldwide, it would be helpful to try implement training for large-group teaching. 

Based on our findings and similar studies, there is a positive relationship between the PAL program and the improvement of chest tube insertion technical skills on surgical residents. Accordingly, the PAL program can increase knowledge and technical of chest tube insertion in surgical residents. Therefore, adding the PAL program to the surgical residency curriculum is recommended. Our limitation must be acknowledged that we had limited participants due to the limited number of residents in each year. Participants, especially those who entered the study one month after admission were on different competency levels and knowledge about chest tube insertion.

## Declaration

### Ethics approval and consent to participate:

The study protocol was approved by the institutional review board and the ethics committee of shiraz university of medical science (IR.SUMS.REC.1399.1209).

### Consent for publication:

All authors agree with the publication of this article.

### Conflict of interest:

The authors declare that they have no conflict of interest.

### Funding:

This research was funded by the Rajaee (Emtiaz) trauma research center.

### Authors’ contributions:

I. Deilami: data gathering, workshop teaching; M. Amini: data analysis, study design; S. Paydar: study design, data gathering; HR. Abbassi: editing; S. Bolandparvaz: editing.

### Acknowledgments

This article is based on the residency thesis with the project no: 22177 of Shiraz University of Medical Sciences, Shiraz, Iran.
